# Correction: Surface modification of graphene with functionalized carbenes and their applications in the sensing of toxic gases: a DFT study

**DOI:** 10.1039/d3ra90067c

**Published:** 2023-07-31

**Authors:** Sarah Aldulaijan, Afnan M. Ajeebi, Abdesslem Jedidi, Sabri Messaoudi, Noureddine Raouafi, Adnene Dhouib

**Affiliations:** a Chemistry Department, College of Science, Imam Abdulrahman Bin Faisal University P.O. Box 1982 Dammam 31441 Saudi Arabia saaldulaijan@iau.edu.sa afnanajeebi@hotmail.com amdhouib@iau.edu.sa; b Chemistry Department, Faculty of Science, King Abdulaziz University Jeddah 21589 Saudi Arabia ajedidi@kau.edu.sa; c Laboratoire des Matériaux Molécules et Applications, Université Tunis Carthage, IPEST La Marsa 2070 Tunisia; d Department of Chemistry, College of Science, Qassim University Buraidah 51452 Saudi Arabia S.messaoudi@qu.edu.sa; e Sensors and Biosensors Group, Laboratory of Analytical Chemistry & Electrochemistry (LR99ES15), Faculty of Science, University of Tunis El Manar 2092 Tunis El Manar Tunisia noureddine.raouafi@fst.utm.tn

## Abstract

Correction for ‘Surface modification of graphene with functionalized carbenes and their applications in the sensing of toxic gases: a DFT study’ by Sarah Aldulaijan *et al.*, *RSC Adv.*, 2023, **13**, 19607–19616, https://doi.org/10.1039/D3RA02557H.

The authors regret the omission of a funding acknowledgement in the original article. This acknowledgement is given below.

This research was funded by the Deanship of Scientific Research, Imam Abdulrahman Bin Faisal University (ref. 2021-019-Sci). For computer time, this research (ref. k1495) used the resources of the Supercomputing Laboratory at King Abdullah University of Science & Technology (KAUST) in Thuwal, Saudi Arabia.

The Royal Society of Chemistry apologises for these errors and any consequent inconvenience to authors and readers.

## Supplementary Material

